# mRNA 3′ End Processing Factors: A Phylogenetic Comparison

**DOI:** 10.1155/2012/876893

**Published:** 2012-02-06

**Authors:** Sarah K. Darmon, Carol S. Lutz

**Affiliations:** Department of Biochemistry and Molecular Biology and Graduate School of Biomedical Sciences, UMDNJ-New Jersey Medical School, Newark, NJ 07103, USA

## Abstract

Almost all eukaryotic mRNAs possess 3′ ends with a polyadenylate (poly(A)) tail. This poly(A) tail is not encoded in the genome but is added by the process of polyadenylation. Polyadenylation is a two-step process, and this process is accomplished by multisubunit protein factors. Here, we comprehensively compare the protein machinery responsible for polyadenylation of mRNAs across many evolutionary divergent species, and we have found these protein factors to be remarkably conserved in nature. These data suggest that polyadenylation of mRNAs is an ancient process.

## 1. Introduction

Almost all eukaryotic mRNAs have a poly(A) tail at their 3′ ends, with the most notable exception being histone mRNAs. The process by which mRNAs acquire a poly(A) tail is termed polyadenylation. Polyadenylation is a tightly coupled, two-step process that first endonucleolytically cleaves the pre-mRNA and subsequently adds an unencoded poly(A) tail (reviewed in [1–7]). Poly(A) tails serve the mRNA in many ways, aiding in mRNA translation, facilitating transport from the nucleus to the cytoplasm, and promoting stability [8–12]. The addition of the poly(A) tail is a highly coordinated event, requiring cooperation from both *cis*-acting RNA sequence elements and *trans*-acting protein factors to complete the process [13, 14]. Alternative or regulated polyadenylation likely requires further cooperation and integration of efforts.

**Figure 1:**
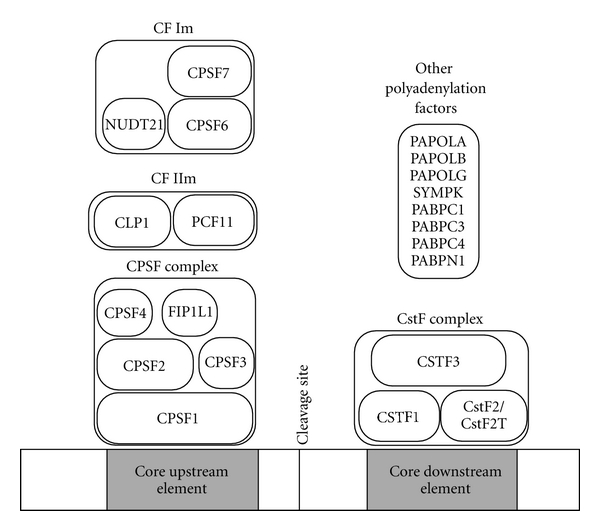
Human polyadenylation factors. Human basal polyadenylation factors are composed of many multisubunit complexes: CPSF, CstF, CFIm, and CFIIm. There are also many other auxiliary factors that contribute to polyadenylation; representative factors are listed at the top right.

 Two sequence elements in mammals serve as the core polyadenylation elements: the AAUAAA or a variant, and a U/GU-rich element located downstream 10–30 nts of the actual site of polyadenylation (Figure 1, [15, 16] and references therein). The cleavage site, where the poly(A) tail is added, is located in between these two sequence elements and is often a CA dinucleotide, but it has some variability ([15] and references therein). The AAUAAA element serves as a binding site for the CPSF (cleavage and polyadenylation specificity factor) complex, a complex of four subunits, while the U/GU-rich element binds the CstF (cleavage stimulation factor) complex, a trimeric complex of proteins (Figure 1). Yeast polyadenylation signals have a slightly different composition but bind similar protein complexes with slightly different orientation.

The protein factors that make up the basal polyadenylation machinery in mammalian cells were purified, isolated, and cloned by many laboratories in the 1990s (including [17–23]). Additional proteins that influence or regulate polyadenylation have also been identified over the past decade or more (including [24–27]). Many of the basal polyadenylation factors from mammalian cells, and some additional factors, have been shown to have orthologues or homologs in other organisms. A report has compared the mammalian polyadenylation machinery with that of the protozoan *Entamoeba histolytica* [27]; however, no comprehensive study has been undertaken to compare and contrast the polyadenylation machinery from a number of different species. Here, we have compared basal polyadenylation factors from human to species ranging from mouse to plants and archaea and have found most of them to be remarkably conserved. These findings are consistent with the universal eukaryotic nature of mRNAs having a poly(A) tail.

## 2. Materials and Methods

### 2.1. Homologous Human Polyadenylation Factors

The human polyadenylation factors were compared to 14 different species that are shown in Table 1. Using the NCBI protein-protein BLAST (blastp, version 2.2.25), we compared the human polyadenylation factor protein sequences to homologous sequences present in the other species through the nonredundant database (nr). The highest ranked protein with a bit score of 50 or greater was chosen as the homolog. These proteins were compared to the human factor in question by the number of amino acids present in the homolog relative to the human factor, as well as by amino acid alignment of the same or similar amino acids.

### 2.2. Domain Comparison

The NCBI conserved domain database was used to find the domains in each of the human polyadenylation factor proteins as well as known published human domains. The presence of these domains was determined in each of its corresponding homologs. The domains were aligned using the same parameters of comparison as the whole protein comparison.

## 3. Results and Discussion

By comparing basal polyadenylation factors from a phylogenetic perspective, we can gain insight into functional and mechanistic differences that may exist in different species. We have compared and contrasted polyadenylation factors from a number of different species for their overall homology and percent identity relative to human, as well as for their similarity in specific protein domains. The species we analyzed from mouse to archaea are shown in Table 1. Tables 2 and 3 show the specific locus name for a given polyadenylation factor for each species. In some instances, the locus name may not reveal much. CPSF 1, 2, 3, and 4 are also known as CPSF 160, 100, 73, and 30, respectively. CSTF 1, 2, and 3 are known as CstF 55, 64, and 77, respectively; CPSF 6 is also known as CFIm68; PAPOLA is poly(A) polymerase.

 Human polyadenylation factor homologs were found for most of the species with the major exception of archaea and yeast (Tables 2 and 3). Archaea only had homologs in the CPSF complex. A polymer “A” tail is not found in *H. volcanii* [28]. In some archaea, a random copolymer tail is added by the exosome or PnPase [29]. Therefore, most of the human polyadenylation factors evolved after archaea.

 Both yeast species did not contain homologs for the entire CFIm complex and CSTF1 (Table 3). This emphasizes a major difference in yeast and human polyadenylation (reviewed in [1, 13]). CFIm is involved in early steps of polyadenylation and recruits other polyadenylation factors [14, 30, 31]. This is achieved by NUDT21 binding to a UGUA sequence [32]. The Hrp1p complex in yeast likely plays a similar role as CFIm. Hrp1p binds to the polyadenylation enhancer element [33] and interacts with RNA14 and RNA15 [34]. RNA14 and RNA15 are homologs of the CSTF2 and CSTF3 human proteins. Therefore, Hrp1p may abrogate the need for CSTF1 and CFIm complex in yeast.

 The malaria mosquito (*Anopheles gambiae*) did not contain any poly(A) polymerase homologs (Table 2). This is most likely due to missing gene annotation because the yellow fever mosquito (*Aedes aegypti*) and southern house mosquito (*Culex quinquefasciatus) *contain a poly(A) polymerase homolog.

 Humans have gene variant forms of CSTF2, PABPC, and PAPOLA that are tissue-specific. CSTF2T (CstF-64 tau) is expressed in the testis and brain and is found in meiotic and postmeiotic germ cells where CSTF2 is inactivated [35]. This variant was only found in the human and mouse species. Cytoplasmic PABP has two cell-specific isoforms, PABPC3 and PABPC4. PABPC3 is found in the testis and has a lower binding affinity to RNA [36], and PABPC4 is inducible in T cells [37]. Both of these proteins are found in mouse and the eudicot plants. PABPC4 is also found in chicken, trypanosomes, and eudicot plants. Poly(A) polymerase has a testis-specific gene variant form, PAPOLB [38]. Homologs are also found in mouse and plants. PAPOLG homolog was only found in mouse. The human gene variant homologs of PABPC and PAPOLA found in plants emphasize the difference in plant and human polyadenylation (reviewed in [39]). Thale cress contains at least eight isoforms of PABP and four isoforms of PAP [40, 41]. Homologs for most tissue-specific human polyadenylation factors are more recently evolved since homologs are only found in mouse.

 Humans have several isoforms of the polyadenylation factors FIPI1L, CSTF1, and CSTF3 (Tables 2 and 3). Multiple isoforms of these factors were not found in any of the other species. The NUDT21 complex contained the most evolutionary conserved multiple isoforms with isoforms only in *Drosophila*, *T. cruzi*, and eudicots. *Drosophila *has the most species-specific isoforms for human factors CPSF1, CPSF2, CSTF1, NUDT21, and PAPOLA, but there is generally only one isoform of these factors in the other species. Therefore, isoforms of some polyadenylation factors are not evolutionary conserved and often their function is species specific.

 We concluded from this comparison that human basal polyadenylation factors are quite well conserved evolutionarily with the exceptions of archaea and some yeast factors, tissue-specific gene variants, and protein isoforms.

 We next further analyzed the identified homologs of the human polyadenylation factor protein sequences to see how stringently the factors were conserved by two different means: conservation of protein length and conservation of the amino acids in the alignment with the same or a similar amino acid (Table 4). These analyses were performed using the NCBI databases and BLAST alignment tools.

**Figure 2:**
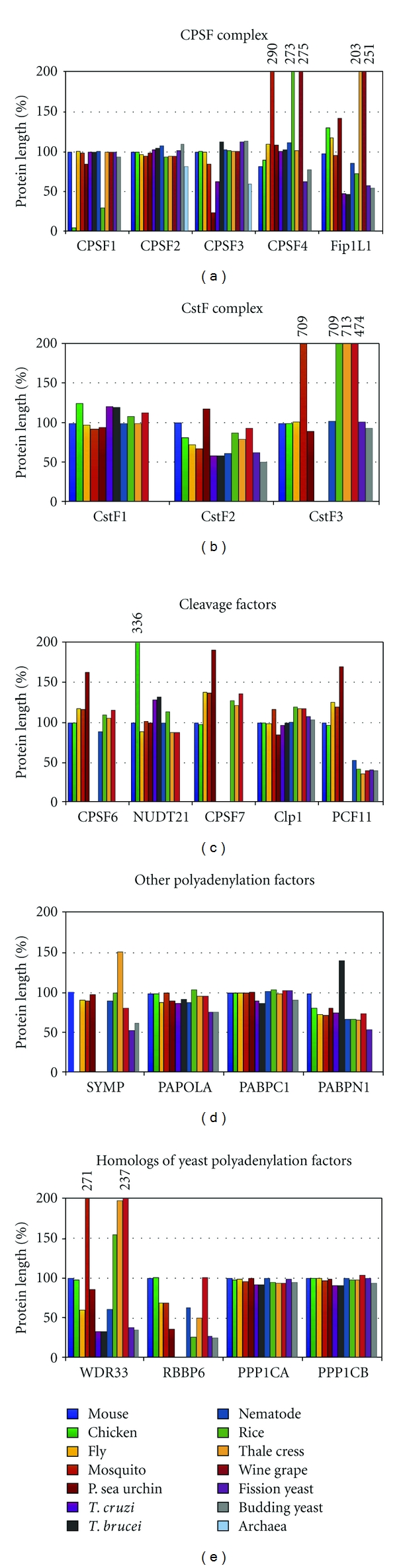
Protein length changes in polyadenylation factors. The changes in length of polyadenylation factors between homologs were compared to the human homolog of each specific polyadenylation factor. See the top left for color code of species. If the homolog was greater than 2 times the length of the human protein, the value is given above the bar.

 Protein length can change through evolution by many mechanisms, including insertions, deletions, and transposable elements. The general belief is that protein length increases through evolution [42]. While there tends to be a protein lengthening from *E. coli* to yeast, nematode, and humans, species of fungi, animals, and plants tend to have a conservation of protein length [43]. The majority of the polyadenylation factor homologs remained within 20% of the same size as the corresponding human polyadenylation factor (Figure 2). CSTF2, FIP1L1, and PABPN1 shortened as the species became evolutionary more diverse and the yeast homologs are ~50% of the size of their human counterparts. The PCF11 protein length was relatively conserved evolutionary down to purple sea urchin but nematode, plants, and yeast homologs are only half the size of the human protein.

 There are specific species that do not follow the evolutionary trends. In insects, purple sea urchin, and plants, the protein lengths of the homologs tend to increase in size dramatically when protein length is not conserved. CSTF3 homologs in plants and mosquito are seven times larger than the human protein. While more uncommon, there are some truncated proteins within these species. For example, the CPSF1 homolog in rice and the CPSF3 homolog in purple sea urchin are ~25% of the human protein length (Figure 2). 

 The protein length of the chicken homologs of CPSF1 and NUDT21 provides evidence for some errors in the species gene annotation. The chicken CPSF1 homolog is only 5% of the length of human CPSF1 (Figure 2) and is not large enough to be a functional human homolog. Zebra finch (*Taeniopygia guttata*) and wild turkey (*Meleagris gallopavo*) have CPSF1 homologs that were about 75% the size of the human protein (data not shown). Therefore, it is likely that the chicken CPSF1 gene annotation is incorrect. The chicken NUDT21 homolog is three times larger than the human homolog. The zebra finch (*Taeniopygia guttata*) NUDT21 homolog is 110% the size of the human protein length. The chicken autocrine motility factor receptor (AMFR) is annotated incorrectly and contains two genes: the human NUDT21 and AMFR human homologs.

 We concluded that while most of the polyadenylation machinery was similar in protein length as compared to the corresponding human proteins, there were some significant differences in either direction in insects, purple sea urchin, and plants. Also, some homologs did show a lengthening trend in proteins through evolution from yeast to human.

**Figure 3:**
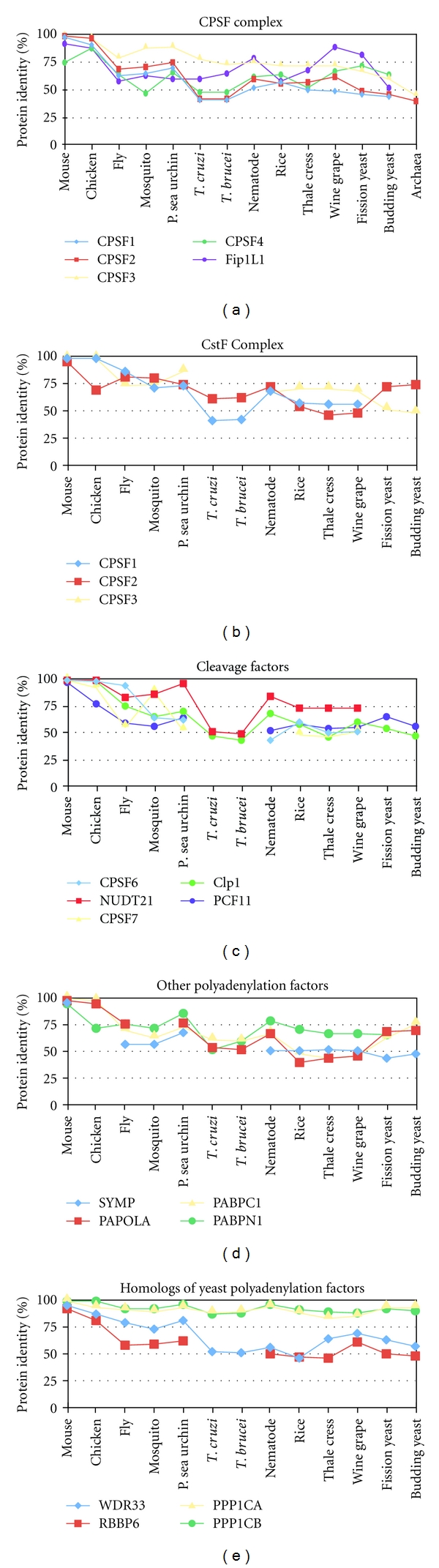
Conservation of protein sequences in polyadenylation factors. The protein sequence for each factor or complex of the human basal polyadenylation machinery was compared to the homologous factors in each species to determine how much of the protein sequence is changed.

 Another way to determine the conservation of polyadenylation factors is to determine how the amino acid sequence has changed through evolution. The protein sequence that aligned to the human polyadenylation factor identity was compared to determine how many amino acids were the same or similar. We performed this analysis by aligning the two protein sequences in NCBI and recording the percent positive. As to be expected, most of the factors decreased in similarity as the comparison was performed from mouse to yeast and plants. Most of the factors retained at least 40% of the human amino acid sequence (Figure 3). PPP1CA and PPP1CB, which are homologous factors of the yeast polyadenylation factor GLC7, were surprisingly the most conserved among all the factors with at least 90% positive identity.

 To further look into the phylogenetic comparison, protein domains present in the human basal polyadenylation factors were compared to the domains present in the homologous factors in other species using the same methods as we used in analyzing the whole protein. This analysis with published human domains can help verify homologs and determine if the polyadenylation factors retain their same function(s) throughout evolution. The same protein domains were found in many, but not all, of the homologous factors.

**Figure 4:**
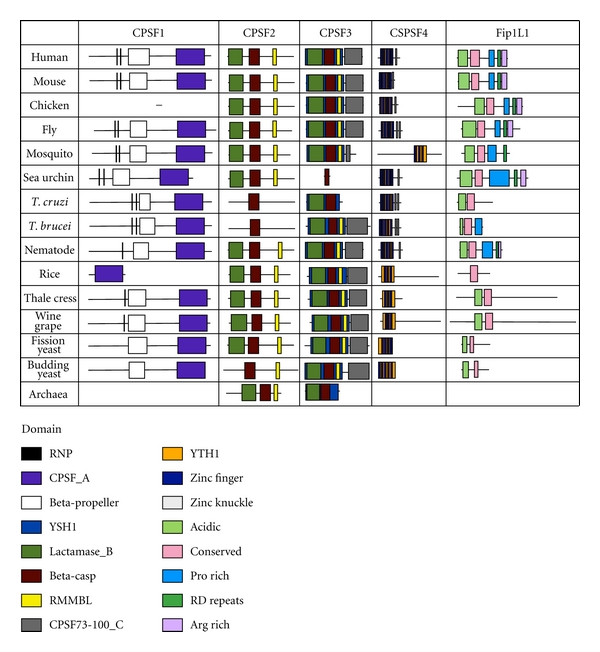
Domain homology of CPSF. Schematics of proteins are shown approximately to scale. The domains were identified by the NCBI conserved domain database or known published domains. CPSF1 contains domains involved in RNA binding: two RNP binding motifs, CPSF_A and beta-propeller domains. CPSF3 contains a YSH1 domain that contains the lactamase, beta-caspase, and RNA-metabolizing metallo-beta-lactamase (RRMBL) domains. CPSF73-100_C is the conserved C-terminal domain of CPSF3. CPSF2 contains the lactamase, beta-caspase, and RRMBL domains. CPSF4 contains a protein-protein interaction YTH1 domain that contains five zinc finger domains. FIP1L1 contains an acidic, conserved, proline-rich, RD repeats and arginine-rich domains involved in protein-protein interactions.

**Figure 5:**
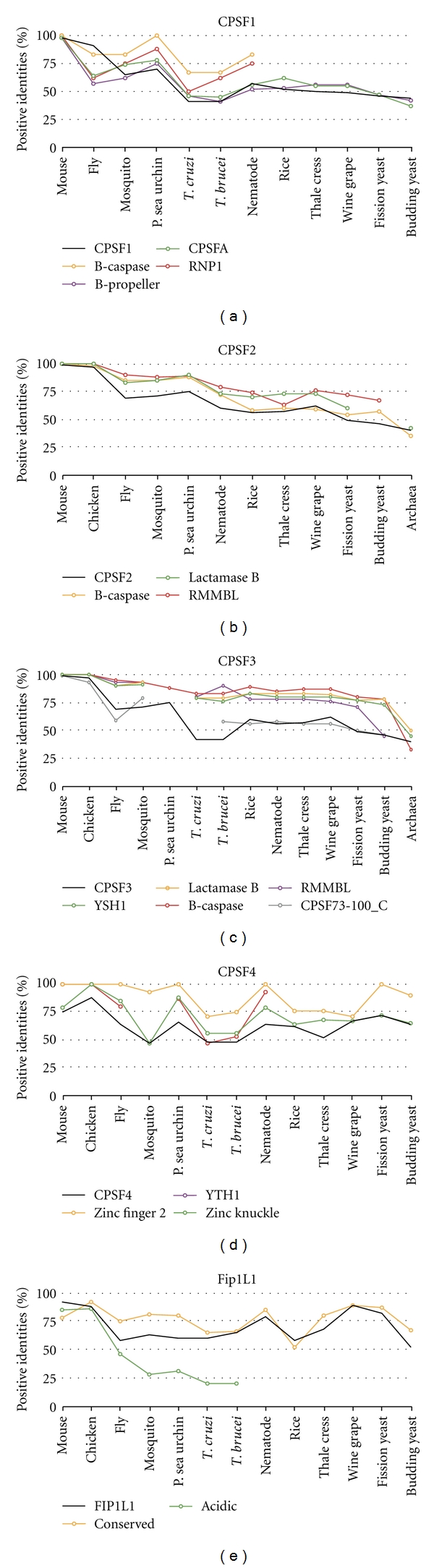
Conservation of protein sequence between the protein domains of the CPSF subunits. The amino acid sequence of human CPSF subunits and domains were compared to the homologous factor protein and domains in other species.

 CPSF1 (CPSF-160) has four domains found in human (Figure 4). The CPSF A domain was found in all the homologous factors. The CPSF A domain is a region that may be involved in RNA/DNA binding but its function is unknown. The beta-propeller domains were found in all the homologs except the truncated rice homolog. The beta-propeller domain contains five propeller repeats and is required for RNA binding in the yeast homolog [44]. Two RNP type binding motifs are present in CPSF1 and may be involved in RNP binding [45]. These motifs were evolutionary conserved down to trypanosome. None of the domains amino acid sequences were more conserved than the entire CPSF1 (Figure 5).

 CPSF3 (CPSF-77) has five highly conserved domains (Figure 4). The YSH1 domain is the yeast homolog of CPSF3 which contains the entire metallo-beta-lactamase domain. Many metallo-beta lactamases are zinc-dependent nucleases [46], and CSPF3 is the predicted pre-mRNA 3′ end processing nuclease [47, 48]. The lactamase B domain contains four out of the five canonical metallo-beta-lactamase sequence motifs. RNA-metabolizing metallo-beta-lactamase (RMMBL) domain contains the fifth motif. B-caspase is a cassette inserted between the fourth and fifth beta-lactamase motifs. The B-caspase and lactamase domains form an interface around the active site [48]. The CPSF73-100_C domain is the conserved C-terminal region of CPSF3. These domains were found in all species examined except the purple sea urchin, Trypanosome (*T. cruzi*), and archaea. These species had missing domains due to the fact that the homologs were truncated. Except for CPSF73-100_C, all of the domains amino acid sequences were more conserved than the entire protein in all species excluding archaea (Figure 5). Therefore, the domains within the CPSF3 protein, except for the sea urchin homolog, may be conserved to maintain the endonuclease function.

 CPSF2 (CPSF-100) is similar to CPSF3 and both proteins share all but one domain (Figure 4). CPSF2 is an inactive nuclease with an inability to bind two zinc molecules [48] and its function is unknown. Trypanosomes are missing the entire metallo-beta lactamase domain. Sequence conservation of these domains is only slightly higher compared to the entire protein (Figure 5).

 The CPSF4 (CPSF-30) protein has YTH1, zinc knuckle, and five zinc finger domains (Figure 4). The YTH1 domain is the yeast homolog of CPSF4 and encompasses all five zinc fingers. This domain was found in all species analyzed. The zinc knuckle CCHC motif aids in binding to polyU RNA [49]. This domain was absent in plants and yeast homologs. Two zinc knuckles are present in trypanosomes and *Drosophila*. Zinc fingers are involved in protein and RNA interactions [50]. All five zinc finger CCCH motifs were found in most of the species examined with four motifs present in fission yeast and three in plants and mosquito homologs. The second zinc finger domain is most conserved in yeast and is lethal when deleted [50]. This conservation was also maintained with at least 90% positive identities in all the species, except trypanosomes and plants which maintain at least 70% positive identity (Figure 5). Yeast homologs have all five zinc finger CCCH motifs; however, excluding the second zinc finger domains, none of the zinc finger domains maintained more than 65% positive identities to human. The zinc knuckle domain (when present) and multiple zinc finger motifs are highly conserved and may maintain the ability of CPSF4 homologs to bind to RNA.

 FIP1L1 has four domains involved in protein-protein interactions, and these domains are present in most species (Figure 4). The acidic domain binds to PAP [51, 52]. An acidic domain was found in all species except rice. The conserved region is found in all the species and interacts with CPSF4 [52]. The pro-rich domain function is unknown but was found to be evolutionary conserved to nematode. The C-terminal portion of FIP1L1 is made up of RD repeats and an arginine-rich region; it binds to CPSF1 and to U-rich RNA [52]. These two domains were found in all species except trypanosomes, plants, and yeast. None of the domains amino acid sequences were conserved more than the entire protein (Figure 5). However, the presence of these domains suggests that the FIP1L1 homologs retain their binding ability to PAP and the CPSF complex, while the interaction of FIP1L1 directly with RNA may be lost in trypanosomes, plants, and yeast.

**Figure 6:**
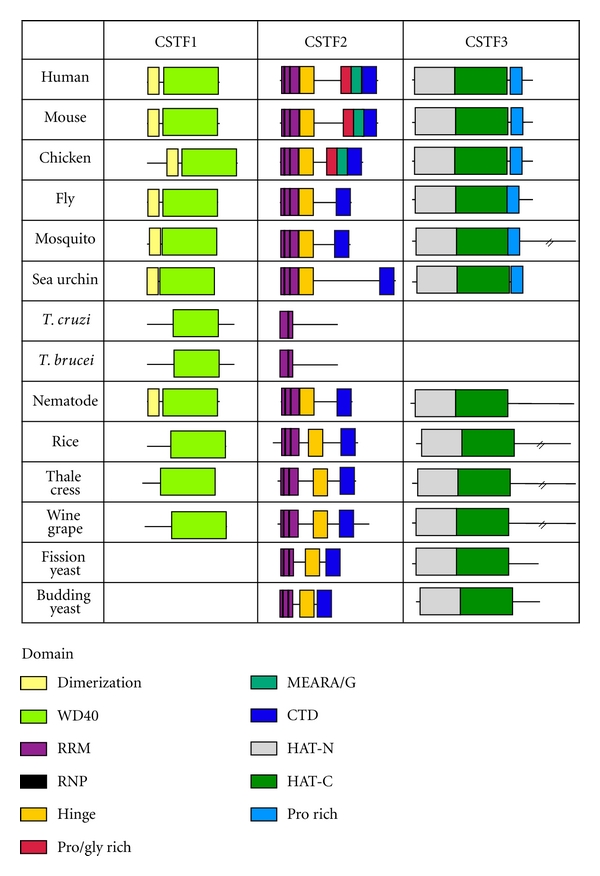
Domain homology of the CstF complex. Schematics of proteins are shown approximately to scale. The domains were identified by the NCBI conserved domain database or known published domains. CSTF1 contains dimerization and WD40 domains involved in protein-protein interactions. CSTF2 has five domains: RRM, hinge, proline/glycine rich, MEARA/G, and CTD. The RRM is involved in CSTF2 RNA binding. The hinge and CTD domains are involved in protein-protein interactions. CSTF3 has three protein interacting domains: HAT-N, HAT-C, and proline-rich domains.

**Figure 7:**
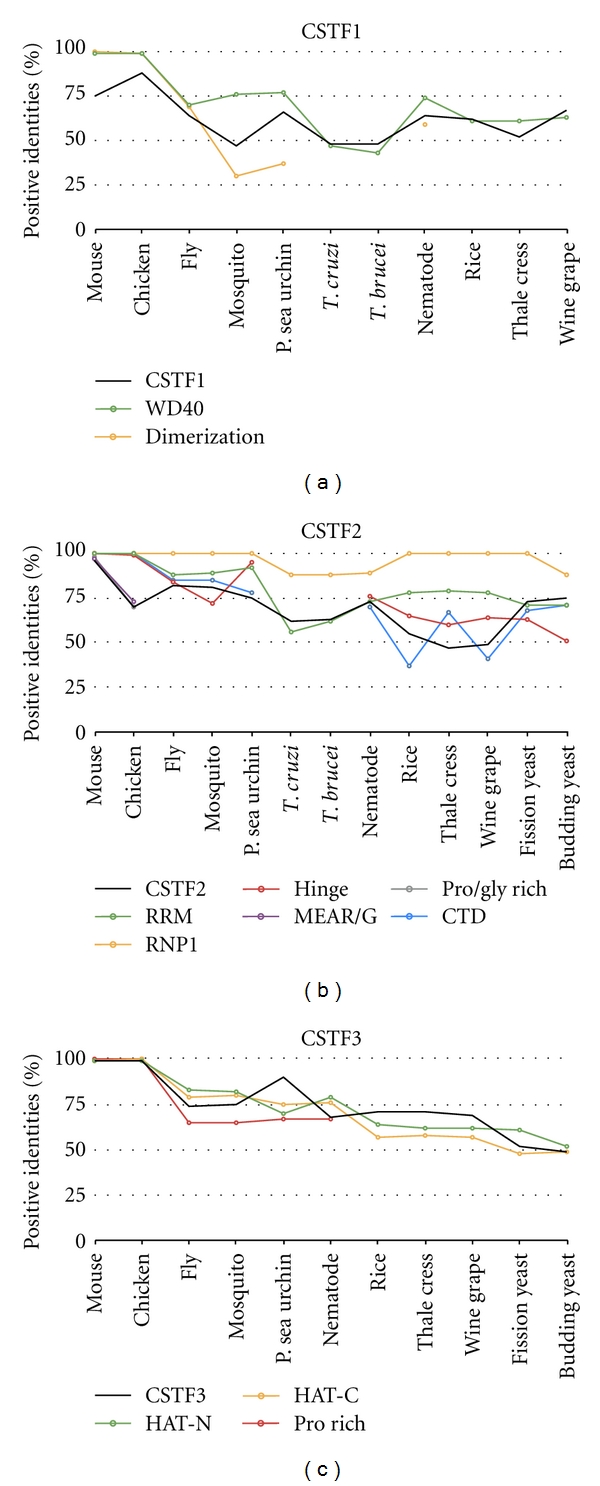
Conservation of protein sequence between the protein domains of the CSTF subunits. The amino acid sequence of human CSTF subunits and domains were compared to the homologous factor protein and domains in other species.

 CSTF1 (CstF-50) has two domains, WD40 and a dimerization domain (Figure 6). The WD40 domain has seven beta-transducin repeats, and deletion of this domain in CSTF1 reduces binding to CSTF3 [53]. This domain was found in all species analyzed. The conservation of amino acids of the domain was similar to the entire protein (Figure 7), but this is most likely due to the domain comprising 75% of the entire protein. The dimerization domain is involved in homodimerization of CSTF1 [53, 54]; this domain can also bind to the CTD of RNA polymerase II (RNA pol II) [55]. The dimerization domain was present in all species except for trypanosomes and plants. Therefore, all the CSTF1 homologs may bind to the CSTF2 homologs or a similar protein. Plants and trypanosome CSTF1 homologs may not self-dimerize or associate with RNA pol II.

 CSTF2 (CstF-64) has five domains: an RNA recognition motif (RRM), hinge, MEARA/G, pro-rich, and CTD domains (Figure 6). The RRM is involved in sequence-specific RNA recognition [53, 56–58]. Within this domain are two RNP binding motifs. All the species examined contained the RRM domain and RNP motifs. Trypanosomes have only the second RNP motif. The RRM domain is conserved more than the entire protein in all species examined except nematode, trypanosomes, and yeast (Figure 7). The hinge domain is involved in protein-protein interactions with CSTF3 and SYMPK [53]. This domain is also involved in nuclear localization [59]. This domain is present in all species examined except trypanosomes, and the domain amino acid sequence is conserved more than the protein in all species except insects and yeast (Figure 7). The CTD domain is a three-helix bundle and involves protein-protein interactions with CSTF2 and PCF11 in the yeast homologs [60]. The CTD domain is found in all species except trypanosomes. Before the CTD domain is a proline/glycine-rich domain (pro-rich) and a 12 repeat MEARA/G domain. The functions of these domains are unknown and they only are present in mouse and chicken homologs. Therefore, CSTF2 homologs may maintain the same functions except for the trypanosome homologs.

 CSTF3 (CstF-77) has three domains: HAT-N, HAT-C, and pro-rich domains (Figure 6). The HAT (half-A-TPR) domain is a variant of the tetratricopeptide repeat (TPR) domain. CSTF3 contains 12 HAT motifs [61]. HAT-N contains motifs 1–5 and HAT-C contains motifs 6–11. The function of the HAT-N domain is unknown. The HAT-C domain is involved in many protein-protein interactions. This includes self-dimerization and interaction with the second beta-propeller motif of CPSF1 [61, 62]. Both HAT-N and HAT-C motifs are found in all species examined. The pro-rich domain interacts with the WD40 region in CSTF1 and the hinge region in CSTF2 [53]. This domain was found to be evolutionary conserved down to purple sea urchin but was not found in plants and yeast (Figure 7). Therefore, most of the CSTF3 homologs may perform the same functions as the human counterparts. Plant and yeast CSTF3 homologs do not have the pro-rich domain and may not associate with CSTF1 and CSTF2 homologs.

**Figure 8:**
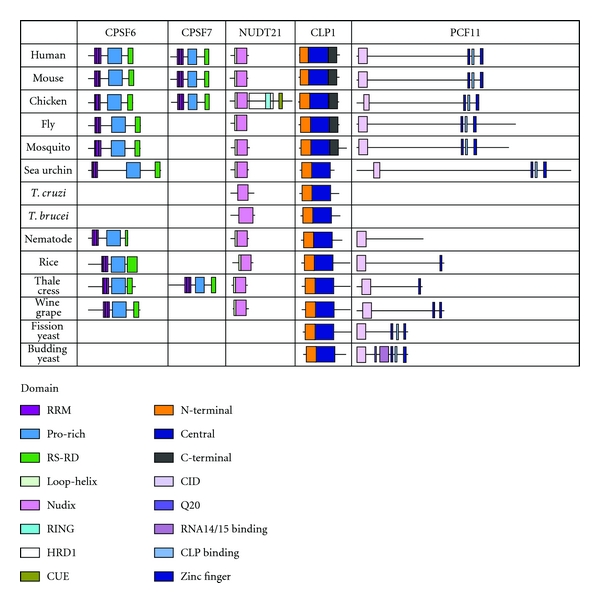
Domain homology of CFIm and CFIIm. Schematics of proteins are shown approximately to scale. The domains were identified by the NCBI conserved domain database or known published domains. CPSF6 contains an RRM, a proline-rich, and RS domains involved in protein-protein interaction. NUDT21 has two domains: a loop-helix domain and a Nudix domain that binds RNA. CLP1 has N-terminal, central, and C-terminal domains. PCF11 has a CTD interacting domain (CID), a Clp binding domain, and two zinc fingers.

**Figure 9:**
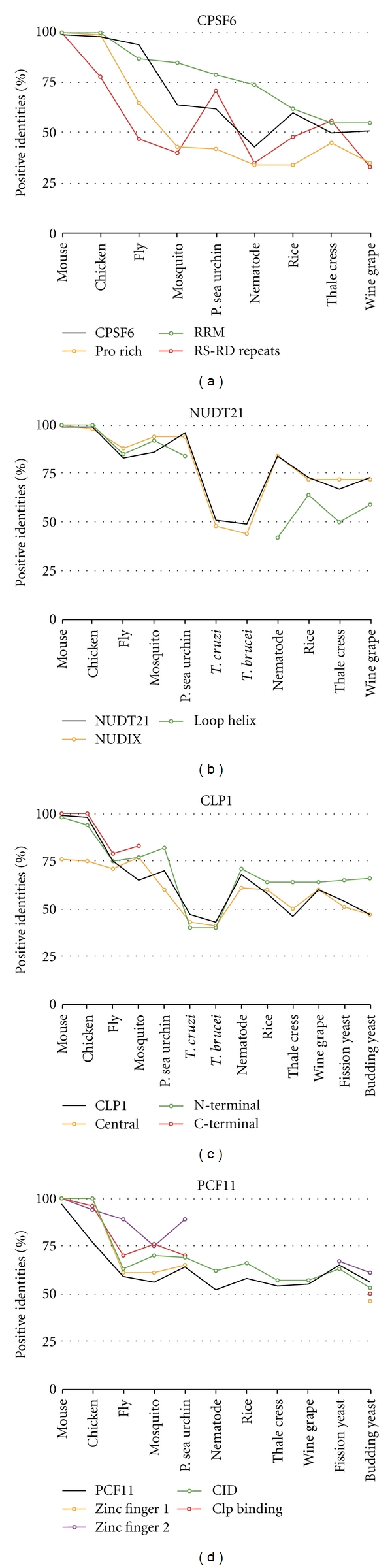
Conservation of protein sequence between the protein domains of the CFIm and CFIIm subunits. The amino acid sequence of human CFIm and CFIIm protein subunits and domains were compared to the homologous factor proteins and domains in other species.

 The CFIm complex domains are very well conserved. CPSF6 (CFIm68) and CPSF7 (CFIm59) are very similar proteins and share their three domains: RRM, proline-rich, and RS domains (Figure 8). These domains were present in all CPSF6 and CPSF7 homologs. The RRM domain was the only domain where the amino acid sequence was more conserved than the entire protein (Figure 9). The RRM domain of CPSF6 does not bind to RNA but is required to bind to NUDT21 [63]. The proline-rich domain may be a weak nuclear localization signal [63]. The RS domain is a dipeptide repeat region of RS, RE, or RD and associates with spliceosomal SR proteins [63, 64]. NUDT21 (CFIm25) has two domains: loop-helix and Nudix domains. These two domains form a complex to bind UGUA RNA sequence elements and eliminate the typical Nudix hydrolase activity [32]. These domains were found in all species except trypanosomes which do not have the loop binding domain. Therefore, the CFIm homologs may form a complex and perform similar functions as the human counterparts.

 CLP1 contains three domains that are not more conserved than the entire protein (Figure 8). The N-terminal and central domains are found in all homologs examined. The C-terminal domain is only conserved evolutionary until insects. The central domain contains the Walker motif which binds ATP/GTP [65]. Clp1 is a kinase involved in tRNA splicing [66]. Therefore, the CLP1 homologs may have the same kinase activity. PCF11 has three domains, CTD interacting domain (CID), CLP1 binding domain (CLP BD), and two zinc fingers. These domains were slightly more conserved than the entire protein (Figure 9). The CID domain is found in all homologs. At least one zinc domain was found in all species except nematode. Clp binding domain was found evolutionary conserved down to sea urchin and yeast. Budding yeast has additional unique features of a Q20 and RNA14/15 binding domain. PCF11 homologs maintain the CTD and some protein-protein interactions.

**Figure 10:**
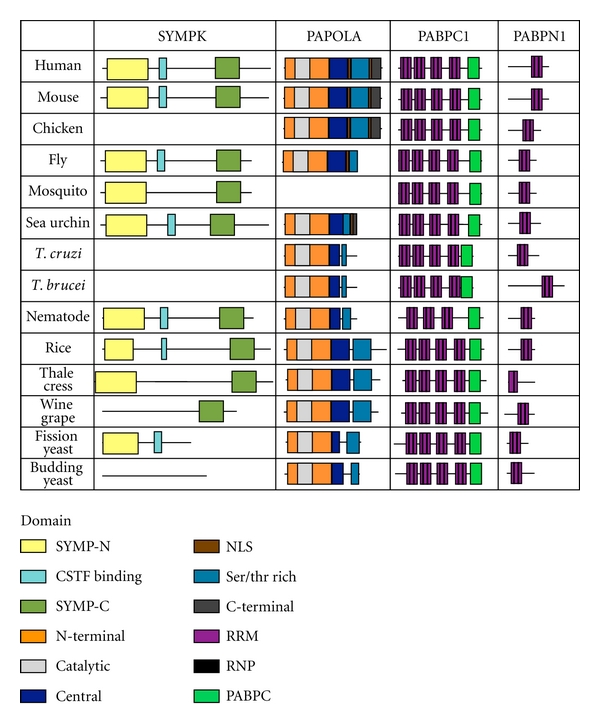
Domain homology of SYMP, PAPOLA, PABPN1, and PABPC4. Schematics of proteins are shown approximately to scale. The domains were identified by the NCBI conserved domain database or known published domains. Symplekin has SYMP-N, SYMP-C, and CstF binding domains. PAPOLA contains many domains including N-terminal, catalytic central, nuclear localization signal (NLS), serine/threonine-rich, and C-terminal domains. The nuclear and cytoplasmic PABP proteins contain RRM domains. PABPC1 has a protein-protein interacting PABC domain.

**Figure 11:**
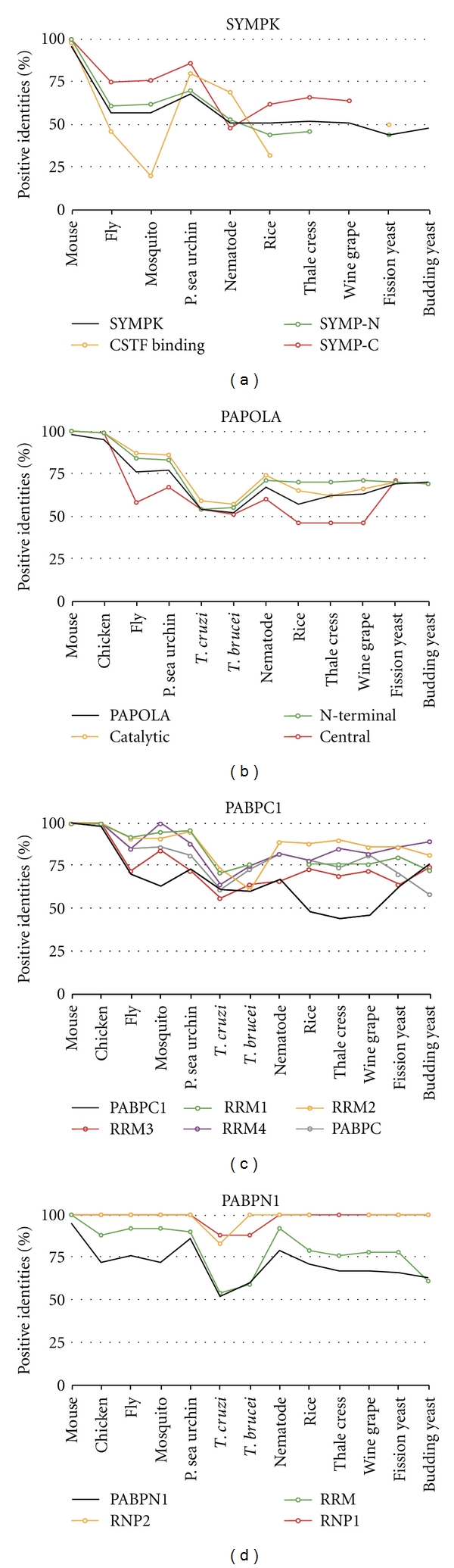
Conservation of protein sequence between the protein domains of SYMP, PAPOLA, PABPN1, and PABPC4. The amino acid sequence of human polyadenylation factor protein and domains were compared to the homologous factor protein and domains in other species.

 The nuclear and cytoplasmic PABP proteins contain well-conserved RRM domains that bind to the poly(A) tail (Figure 10). PABPN1 has one RRM domain that is found in all the homologs. The RNP motifs are found in all species except thale cress. PAPBC1 has four RRM domains but not all of them are required for RNA binding [67]. These domain and RNP binding motifs were found in all species examined. The nematode homolog only contains three RRM domains. PABPC1 also contains a PABPC domain, which includes a MLLE motif and is involved in protein-protein interactions [68, 69]. The PABPC domain was found in all homologs examined. The RRM and PABPC domains are more conserved than the entire protein in all species except for in trypanosomes (Figure 11). Therefore, the PABP homologs may retain the same functions as the human proteins with protein-protein interactions and binding to poly(A) sequences.

 SYMPK has three domains: SYMP-N, SYMP-C, and CstF binding domain, none of which are well conserved (Figure 10). SYMP-N contains HEAT repeats that are involved in protein-protein interactions including Ssu72 [70]. SYMP-N is found in all homologs except for wine grape and budding yeast. The CstF binding domain binds to the hinge region of CSTF2 [71]. This domain was not found in mosquito, eudicots, or budding yeast. SYMP-C contains the domain involved in tight junctions [72]. This domain was found in all species examined except for yeast. Only the SYMP-C domain is more conserved than the entire protein (Figure 11). Therefore, the function of these homologs, especially in budding yeast, may be through different means.

 PAPOLA homologs contain most of the domains except for the C-terminal domain (Figure 10). The domains present are the N-terminal, catalytic, central, NLS, Ser/Thr-rich, and C-terminal domains. None of the domains have an amino acid sequence which is more conserved than the entire protein (Figure 11). The N-terminal domain contains the catalytic domain which is the nucleotidyltransferase [73]. The N-terminal as well as the central domain was conserved in all species. The entire C-terminal domain was only conserved in vertebrates. The Ser/Thr-rich regions are found in all homologs but the amino acid sequence is not conserved per se. This region is involved in protein-protein interactions [74] and can be phosphorylated to affect poly(A) polymerase activity [75]. Therefore, all the homologs may maintain the same polymerase activity as the human PAPOLA.

 Taken together, protein domains present in the basal polyadenylation factors were for the most part very well conserved between species and therefore most likely maintain similar functions as the human polyadenylation factors. 

## 4. Conclusions

Comparison of the protein machinery involved in mRNA 3′ end formation and how this machinery is conserved in a number of representative species reveals that positive selection has been imposed on retaining the salient functional features of most of the factors. Since humans diverged from yeast and plants approximately 1 billion years ago (990 million years ago for *Drosophila* and nematode, 31 million years ago for chicken, and 91 million years ago for mouse), it is apparent that polyadenylation of mRNAs is an ancient process indeed.

## Figures and Tables

**Table 1:** Species included in the phylogenetic comparison. Common and scientific names are included. The common name will be used in the comparison presented here.

Common Name	Scientific Name
Mouse	*Mus musculus*
Chicken	*Gallus gallus*
Fly	*Drosophila melanogaster*
Mosquito	*Anopheles gambiae*
Purple sea urchin	*Strongylocentrotus purpuratus*
Trypanosome	*Trypanosoma brucei*
	*Trypanosoma cruzi*
Nematode	*Caenorhabditis elegans*
Rice	*Oryza sativa*
Thale cress	*Arabidopsis thaliana*
Wine grape	*Vitis vinifera*
Fission yeast	*Schizosaccharomyces pombe*
Budding yeast	*Saccharomyces cerevisiae*
Archaea	*Haloferax volcanii*

**Table 2:** Homologs of human polyadenylation factors in vertebrates, insects, sea urchin, and trypanosomes. Protein sequences of basal polyadenylation factors from human were compared to other species found in Table 1 to find the homologous factors.

	Human	Mouse	Chicken	Fly	Mosquito	Purple sea urchin	Trypanosomes (*T. cruzi*)	Trypanosome (*T. brucei*)
CPSF complex	CPSF1	CPSF1	LOC770075	CPSF160 isoform A	AGAP011340-PA	LOC584773	Tc00.1047053506871.140	Tb11.01.6170
				CPSF160 isoform B				
	CPSF2	CPSF2	CPSF2	CPSF100 isoform A	AGAP002474-PA	LOC582050	Tc00.1047053504109.110	Tb11.03.0910
				CPSF100 isoform B				
	CPSF3	CPSF3	CPSF3	CPSF73	AGAP001224-PA	LOC591455	Tc00.1047053511003.221	Tb927.4.1340
							
	CPSF4 isoform 1	CPSF4	CPSF4	CLP	AGAP005735-PA	LOC765046	Tc00.1047053511555.40	Tb11.01.4600
	CPSF4 isoform 2							
	FIP1L1 isoform 1	FIP1L1	FIP1L1	FIP1	AGAP001514-PA	LOC580164	Tc00.1047053507601.80	Tb927.5.4320
	FIP1L1 isoform 2							
	FIP1L1 isoform 3							

CstF complex	CSTF1 isoform 1	CSTF1	CSTF1	CST-50 isoform A	AGAP002776-PA	LOC582854	Tc00.1047053511365.10	Tb10.61.0570
	CSTF1 isoform 2			CST-50 isoform B				
	CSTF1 isoform 3							
	CSTF2	CSTF2	CSTF2	CSTF-64	AGAP010918-PA	LOC759858	Tc00.1047053506795.10	Tb927.7.3730
	CSTF2T	CSTF2T						
	CSTF3 isoform 1	CSTF3	CSTF3	SU(F)	AGAP003019-PA	LOC582899		
	CSTF3 isoform 2					LOC591939		
	CSTF3 isoform 3							

CF1m	CPSF6	CPSF6	CPSF6	CG7185	AGAP005062-PA	LOC577326		
	CSPF7	CPSF7	CPSF7					
	NUDT21	NUDT21	AMFR	CG3689 isoform B	AGAP007242-PA	LOC579716	Tc00.1047053509509.40	Tb927.7.1620
				CG3689 isoform C			Tc00.1047053508207.220	

CFIIm	CLP1	CLP1	CLP1	CBC	AGAP007701-PA	LOC763581	Tc00.1047053507027.59	Tb927.6.3690
							Tc00.1047053506941.229	
	PCF11	PCF11	PCF11	PCF11	AGAP001271-PA	LOC582414		

Other factors	PAPOLA	PAPOLA	PAPOLA	hrg isoform A		LOC575500	Tc00.1047053506795.50	Tb927.7.3780
				hrg isoform B				
				hrg isoform C				
	PAPOLB	PAPOLB						
	PAPOLG							
	SYMPK	SYMPK		SYM	AGAP002618-PA	SYMPK		

PABP	PABPC1	PABPC1	PABPC1	PABP	AGAP011092-PA	PABP	Tc00.1047053506885.70	Tb09.211.2150
	PABPC3	PABPC6						
	PABPC4	PABPC4	PABPC4				Tc00.1047053506885.70	Tb09.211.2150
	PABPN1	PABPN1	PABPN1	PABP2	AGAP005117-PA	LOC594592	Tc00.1047053511741.40	Tb09.211.4120

Homologs of yeast polyadenylation factors	WDR33	WDR33	WDR33	CG1109	AGAP001362-PA	LOC574793	TC00.1047053511491.140	Tb927.6.1830
	RBBP6	RBBP6	RBBP6	SNAMA	AGAP011217-PA	LOC584197		
	PPP1CA	PPP1CA	PPP1CC	PP1alpha-96A	AGAP011166-PA	LOC586142	Tc00.1047053508815.110	Tb11.01.0450
	PPP1CB	PPP1CB	PPP1CB	PP1alpha-96A	AGAP003114-PA	LOC752338	Tc00.1047053508815.110	Tb11.01.0450

**Table 3:** Homologs of human polyadenylation factors in nematode, plants, yeast, and archaea. Protein sequences of basal polyadenylation factors from human were compared to other species found in Table 1 to find the homologous factors.

	Human	Nematode	Rice	Thale cress	Wine grape	Fission yeast	Budding yeast	Archaea
CPSF complex	CPSF1	CPSF-1	Os04g0252200	CPSF160	LOC100256706	CFT1	CFT1p	
							
	CPSF2	CPSF-2	Os09g0569400	CPSF100	LOC100267865	CFT2	CFT2p	EPF1
							
	CPSF3	CPSF-3	Os03g0852900	CPSF73-I	LOC100261042	YSH1	YSH1	EPF2
				CPSF73-II				
	CPSF4 isoform 1	CPSF-4	Os06g0677700	CPSF30	LOC100253258	YTH1	YTH1	
	CPSF4 isoform 2							
	FIP1L1 isoform 1	F32D1.9	Os01g0377500	FIP1[V]	LOC100251960	SPAC22G7.10	Fip1p	
	FIP1L1 isoform 2							
	FIP1L1 isoform 3							

CstF complex	CSTF1 isoform 1	CPF-1	Os03g0754900	AT5G60940	LOC100267233			
	CSTF1 isoform 2							
	CSTF1 isoform 3							
	CSTF2	CPF-2	Os11g0176100	CSTF64	LOC100256296	CTF1	RNA15	
	CSTF2T							
	CSTF3 isoform 1	SUF-1	Os12g0571900	CSTF77	LOC100262033	RNA14	RNA14	
	CSTF3 isoform 2							
	CSTF3 isoform 3							

CF1m	CPSF6	D1046.1	Os09g0476100	AT5G55670	LOC100268141			
	CSPF7			AT1G13190				
	NUDT21	F43G9.5	Os04g0683100	AT4G25550	LOC100261950 isoform 1			
				CFIIM-25	LOC100261950 isoform 2			

CFIIm	CLP1	F59A2.4	Os02g0217500	CLPS5	LOC100242380	SPAC22H10.05c	Clp1p	
							
	PCF11	R144.2	Os09g0566100	PCFS4	LOC100251089	SPAC4G9.04c	PCF11	

Other factors	PAPOLA	Pap-1	Os06g0319600	PAPS1	LOC100252483	Pla1	Pap1	
							
							
	PAPOLB		Os06g0558700	PAPS2	LOC100263460			
	PAPOLG							
	SYMPK	F25G6.2	Os07g0693900	ESP4	LOC100266091	PTA1	PTA1	

PABP	PABPC1	PAB-1	Os08g0314800	PAB2	LOC100262903	PABP	PAB1	
	PABPC3			PABP5	LOC100255846			
	PABPC4			PAB5	LOC100255846			
	PABPN1	PABP-2	Os06g0219600	AT5G10350	LOC100242522	PAB2	SGN1	

Homologs of yeast polyadenylation factors	WDR33	R06A4.9	Os04g0599800	FY	LOC100263567	PFS2	PFS2	
	RBBP6	TAG-214	Os10g0431000	AT5G47430	LOC100252571	SPBP8B7.15c	MPE1	
	PPP1CA	GSP-2	OS03g0268000	TOPP7	LOC100256994	DIS2	GLC7	
	PPP1CB	GSP-1	Os06g0164100	TOPP4	LOC100258649	DIS2	GLC7	

**Table 4:** Phylogenetic comparison of human basal polyadenylation factors. Human basal polyadenylation factors were compared to homologous factors in other species by two criteria: percent length is the change in the number of amino acids as compared to the specific human polyadenylation factor. Positive identity is the percentage of amino acids that align to the human polyadenylation factor that are the same or similar to amino acids.

	Species	Homolog	% length	% positive Identity
CPSF1	Mouse	CPSF1	100	98
	Chicken	LOC770075	5	91
	Fly	CPSF160 iso. A	101	63
		CPSF160 iso. B	98	61
	Mosquito	AGAP011340-PA	99	65
	Purple sea urchin	LOC584773	85	70
	Trypanosome (*T. cruzi*)	Tc00.1047053506871.140	100	41
	Trypanosome (*T. brucei*)	Tb11.01.6170	100	41
	Nematode	Cpsf-1	101	52
	Rice	Os04g0252200	30	57
	Thale cress	CPSF160	100	50
	Wine grape	LOC100256706	100	49
	Fission yeast	CTF1	100	46
	Budding yeast	CTF1	94	44

CPSF2	Mouse	CPSF2	100	99
	Chicken	CPSF2	100	97
	Fly	CPSF100 iso. A	97	69
		CPSF100 iso. B	85	68
	Mosquito	AGAP002474-PA	95	71
	Purple sea urchin	LOC582050	99	75
	Trypanosome (*T. cruzi*)	Tc00.1047053504109.110	103	42
	Trypanosome (*T. brucei*)	Tb11.03.0910	105	42
	Nematode	CPSF-2	108	60
	Rice	Os09g0569400	94	56
	Thale cress	CPSF100	95	57
	Wine grape	LOC100267865	95	62
	Fission yeast	CFT2	102	49
	Budding yeast	CFT2	110	46
	Archaea (*H. volcanii*)	EPF1	82	40

CPSF3	Mouse	CPSF3	100	99
	Chicken	CSPF3	101	97
	Fly	CPSF73	100	79
	Mosquito	AGAP001224-PA	85	88
	Purple sea urchin	LOC591455	24	89
	Trypanosome (*T. cruzi*)	Tc00.1047053511003.221	63	78
	Trypanosome (*T. brucei*)	Tb927.4.1340	113	73
	Nematode	CPSF-3	103	75
	Rice	Os03g0852900	102	72
	Thale cress	CPSF73-I	101	72
		CPSF73-II	90	72
	Wine grape	LOC100261042	101	72
	Fission yeast	YSH1	113	67
	Budding yeast	YSH1	114	60
	Archaea	EPF2	60	45

CPSF4	Mouse	CPSF4	82	75
	Chicken	CPSF4	90	88
	Fly	Clp	110	64
	Mosquito	AGAP005735-PA	290	47
	Purple sea urchin	LOC765046	109	66
	Trypanosome (*T. cruzi*)	Tc00.1047053511555.40	101	48
	Trypanosome (*T. brucei*)	Tb11.01.4600	103	48
	Nematode	CPSF-4	112	62
	Rice	Os06g0677700	273	64
	Thale cress	CPSF30	102	52
	Wine grape	LOC100253258	275	67
	Fission yeast	YTH1	63	72
	Budding yeast	Yth1p	78	64

FIP1L1	Mouse	FIP1L	98	92
	Chicken	FIP1L	130	88
	Fly	FIP1	118	58
	Mosquito	AGAP001514-PA	96	63
	Purple sea urchin	LOC580164	142	60
	Trypanosome (*T. cruzi*)	Tc00.1047053507601.80	48	60
	Trypanosome (*T. brucei*)	Tb927.5.4320	47	65
	Nematode	F32D1.9	86	79
	Rice	Os01g0377500	73	58
	Thale cress	FIP1[V]	203	68
	Wine grape	LOC100251960	251	89
	Fission yeast	SPAC22G7.10	58	82
	Budding yeast	Fip1	55	52

CstF1	Mouse	Cstf1	100	99
	Chicken	Cstf1	125	99
	Fly	CstF-50 isoform A	98	87
		CstF-50 isoform B	74	65
	Mosquito	AGAP002776-PA	93	72
	Purple sea urchin	LOC582854	95	74
	Trypanosome (*T. cruzi*)	Tc00.1047053511365.10	121	42
	Trypanosome (*T. brucei*)	Tb10.61.0570	120	43
	Nematode	cpf-1	100	69
	Rice	Os03g0754900	109	58
	Thale cress	AT5G60940	100	57
	Wine grape	LOC100267233	113	57

CstF2	Mouse	CSTF2	101	96
	Chicken	CSTF2	82	70
	Fly	CstF-64	73	82
	Mosquito	AGAP010918-PA	68	81
	Purple sea urchin	LOC759858	118	75
	Trypanosome (*T. cruzi*)	Tc00.1047053506795.10	59	62
	Trypanosome (*T. brucei*)	Tb927.7.3730	59	63
	Nematode	cpf-2	62	73
	Rice	OSs11g0176100	88	55
	Thale cress	CSFF64	80	47
	Wine grape	LOC100256296	94	49
	Fission yeast	CFT1	63	73
	Budding yeast	RNA15	51	75

CstF2T	Mouse	CSTF2t	103	93

CstF3	Mouse	Cstf3	100	99
	Chicken	Cstf3	100	99
	Fly	su(f)	102	74
	Mosquito	AGAP003019-PA	710	75
	Purple sea urchin	LOC591939	78	87
		LOC582899	90	74
	Nematode	Suf-1	103	68
	Rice	Os12g0571900	709	71
	Thale cress	CSTF77	713	71
	Wine grape	LOC100262033	747	69
	Fission yeast	RNA14	102	52
	Budding yeast	RNA14	94	49

CPSF6	Mouse	CPSF6	100	99
	Chicken	CPSF6	100	98
	Fly	CG7185	118	94
	Mosquito	AGAP005062-PA	117	64
	Purple sea urchin	LOC577326	163	62
	Nematode	D1046.1	89	43
	Rice	Os09g0475100	110	60
	Thale cress	AT5G55670	106	50
	Wine grape	LOC100268141	116	51

CPSF7	Mouse	CPSF7	100	99
	Chicken	CPSF7	98	92
	Thale cress	AT1G13190	122	46

NUDT21	Mouse	NUDT21	100	99
	Chicken	AMFR	336	99
	Fly	CG3689 isoform B	89	83
		CG3689 isoform C	104	85
	Mosquito	AGAP007242-PA	102	86
	Purple sea urchin	LOC579716	100	96
	Trypanosome (*T. cruzi*)	Tc00.1047053509509.40	129	51
		Tc00.1047053508207.220	129	51
	Trypanosome (*T. brucei*)	Tb927.7.1620	132	49
	Nematode	F43G9.5	100	84
	Rice	Os04g0683100	114	73
	Thale cress	AT4G25550	88	73
		CFIM-25	98	67
	Wine grape	LOC100261950 isoform 1	88	73
		LOC100261950 isoform 2	92	70

Clp1	Mouse	Clp1	100	99
	Chicken	Clp1	100	98
	Fly	cbc	99	75
	Mosquito	AGAP007701-PA	117	65
	Purple sea urchin	LOC763581	85	70
	Trypanosome (*T. cruzi*)	Tc00.1047053507027.59	97	47
		Tc00.1047053506941.229	97	47
	Trypanosome (*T. brucei*)	Tb927.6.3690	100	43
	Nematode	F59A2.4	101	68
	Rice	Os02g0217500	120	58
	Thale cress	CLPS5	118	46
		CLPS3	123	60
	Wine grape	LOC100242380	118	60
	Fission yeast	SPAC22H10.05c	108	54
	Budding yeast	Clp	104	47

PCF11	Mouse	PCF11	100	97
	Chicken	PCF11	97	77
	Fly	PCF11	126	59
	Mosquito	AGAP001271-PA	120	56
	Purple sea urchin	LOC582414	170	64
	Nematode	R144.2	53	52
	Rice	Os09g0566100	69	58
	Thale cress	PCFS4	52	54
	Wine grape	LOC100251089	70	55
	Fission yeast	SPAC4G9.04c	41	65
	Budding yeast	PCF11	40	56

WDR33	Mouse	WDR33	100	96
	Chicken	WDR33	98	88
	Fly	CG1109	60	80
	Mosquito	AGAP001362-PA	271	74
	Purple sea urchin	LOC574793	86	82
	Trypanosome (*T. cruzi*)	Tc00.1047053511491.140	33	53
	Trypanosome (*T. brucei*)	Tb927.6.1830	33	52
	Nematode	R06A4.9	61	57
	Rice	Os04g0599800	155	47
	Thale cress	FY	198	65
	Wine grape	LOC100263567	237	70
	Fission yeast	PFS2	38	64
	Budding yeast	PFS2	35	58

RBBP6	Mouse	RBBP6	100	93
	Chicken	RBBP6	101	82
	Fly	SNAMA	69	59
	Mosquito	AGAP011217-PA	69	60
	Purple sea urchin	LOC584197	36	63
	Nematode	TAG-214	63	51
	Rice	Os10g0431000	26	48
	Thale cress	AT5G47430	50	47
	Wine grape	LOC100252571	101	62
	Fission yeast	SPBP8B7.15c	27	51
	Budding yeast	MPE1	25	49

PPP1CA	Mouse	PPP1CA	100	100
	Chicken	PPP1CC	98	94
	Fly	PP1alpha-96A	99	92
	Mosquito	AGAP011166-PA	96	90
	Purple sea urchin	LOC586142	100	94
	Trypanosome (*T. cruzi*)	Tc00.1047053508815.110	92	89
	Trypanosome (*T. brucei*)	Tb11.01.0450	92	90
	Nematode	GSP-2	100	95
	Rice	OS03g0268000	95	89
	Thale cress	TOPP7	94	84
	Wine grape	LOC100256994	94	86
	Fission yeast	DIS2	99	94
	Budding yeast	GLC7	95	94

PPP1CB	Mouse	PPP1CB	100	100
	Chicken	PPP1CB	100	100
	Fly	PP1Alpha-96A	100	93
	Mosquito	AGAP003114-PA	97	93
	Purple sea urchin	LOC752338	99	97
	Trypanosome (*T. cruzi*)	Tc00.1047053508815.110	91	88
	Trypanosome (*T. brucei*)	Tb11.01.0450	91	89
	Nematode	GSP-1	100	97
	Rice	Os06g0164100	98	92
	Thale cress	TOPP4	98	90
	Wine grape	LOC100258649	104	89
	Fission yeast	DIS2	100	93
	Budding yeast	GLC7	94	91
